# Author index to Volume 85

**DOI:** 10.1054/bjoc.2001.2201

**Published:** 2001-12


					Aalders JG 1627
Aas T 74
Abbou CC 1515
Abdulkarim B 2017
Abeliovich D 36
Adami H-O 678, 1317
Adams LM 1403
Adjei AA 484
Adjuvant Breast Cancer Project
Belgium 1
Agelaki S 798
Aguggini S 1106
Agus V 1535
Ahern R 953
A'Hern R 1383
Aherne GW 446
Ahlner-Elmqvist M 1478
Ahmad SA 584
Ahmed A 1667
Aillet G 1831
Aine R 552
Akahira J 1731
Al-Mulla F 692, 1032
Alakhov VY 1987
Ali MS 661
Allen-Mersh TG 1640
Allevi G 1106
Allinen M 209
Alman BA 98
Almonte M 966
Alonso ME 204
Alonso MM 1400
Alquati P 1106
Altman DG 944
Alvarez M 966
Amadori D 845
Amiel J 1412
Amino N 102
Ammatuna M 795
Amos CI 1037
Andersen SN 692
Anderson CD 157
Anderson H 137 (L), 1085
Anderson J 493, 831
Anderson RA 1023
Andersson S 1153
Andi A 863
Ando M 1634
Ando Y 1634
Andreyev HJN 692
Andronicos NM 909
Anissimov YG 157
Anker GB 147
Antalis TM 608
Aoki I 313
Arcangeli A 333
Ardizzoni A 1452
Arends JW 692
Arjona D 204
Armand JP 649
Armstrong BK 668
Arndt V 972
Arnold JA 297
Arnold JM 1351
Arrieta O 1396
Ascoli V 379
Asghar MS 875
Asham E 1759
Asosingh K 1387
ATAC Trialists Group 317
Ataman OU 1113
Atar E 504
Athanasou NA 78
Atherton PJ 152
Atzpodien J 1130
Aw SE 1978
Azuma T 692
Azzarelli A 405, 1535
Babin-Boilettot A 1646
Baccarelli A 1304
Bachelot T 816
Baek MJ 1147
Baglietto L 1838
Bagnardi V 1700
Baguley BC 687, 1219
Balaji V 705
Baldini E 1452
Balint E 1813
Balkwill F 891
Ball HM-L 1102
Ballardini B 1838
Balsari A 1964
Balster DA 266
Bamberg M 1233
Bamberger A-M 546
Bar-Meir S 1368
Barajon I 1964
Bardelmeijer HA 1472
Barker DJP 1680
Barr MP 273
Barthelmes HU 1585
Bartlett JMS 1894
Basset-Seguin N 1883
Batlle A 279, 1794
Batrakova EV 1987
Bauer M 1540
Baxevanis CN 1527
Bayoumy S 1037
Bazan V 692
Beauduin M 1
Bebawy M 1998
Begum N-M 122
Beijnen JH 1472, 1627
Belinky A 504
Belka C 1233
Bell A 1551
Bell JA 1958
Bell S 692
Belli S 379
Bello MJ 204
Benamouzig R 692
Benedetti M 379
Bener A 1675
Benhattar J 692, 1713
Bennewith KL 1577
Benraad ThJ 85
Bensmaine MA 509
Benson C 953
Bentzen SM 1113
Beranek M 692
Berchier MC 1444
Berdel WE 1168
Berger F 721
Berghmans T 1444
Bergkvist L 346, 869
Bergman-Jungestrom M 859
Bergstrom A 984
Berkel HJ 991
Bernardo G 141
Berns EMJJ 538, 1359
Berrino F 785
Berruti A 1106
Bersiga A 1106
Bertani H 1607 (L)
Berthelot J-M 1280
Berthier-Vergnes O 1944
Bertoli G 1106
Bertrand S 518
Besse F 1515
Betri E 1106
Bianchi-Scarra G 836
Biddulph JP 350
Biesma B 1456
Biganzoli E 795
Bigelow CE 727
Bilim V 557, 1119
Birch JM 293
Biroccio A 1914
Biron P 816
Bjellerup P 2004
Bjork P 129
Blackhall FH 1094
Bladstrom A 674
Blakey DC 764
Blamey RW 1958
Blangiardo M 1700
Blanks RG 1289
Blay J-Y 816
Bleiberg H 509
Blomley M 1085
Blouin M-J 428
Boddy AV 23
Boddy SC 1023
Bodis S 2010
Boege F 1585
Boffetta P 678, 1317
Bojunga J 1546
Bolsi G 1106
Boman G 1317
Bonadonna G 490
Bonanni B 1838
Bondy ML 1037
Bonfirraro G 333
Boni L 1452
Boonsong A 1492
Boracchi P 795
Borg A 1201
Borg C 816
Borri P 333
Bos AME 1627
Bosetti C 341
Bostick PJ 1340
Bottini A 1106
Botwood N 139 (L)
Botzlar A 1655
Boucaut KJ 608
Bouchardy C 1251
Bouchier-Hayes DJ 273
Boudioni M 641
Boulos PB 1759
Boulton M 641
Bounacer A 1831
Bourgain C 1592
Bourhis J 2017
Boyd A 741
Boyle JM 293
Braakhuis BJM 630
Braham D 1486
Brakenhoff RH 630
Brandl M 1655
Breivik J 692
Brekken C 1968
Bremer M 850
Brenner B 504
Brenner H 367, 972
Bret-Dibat C 1467
Bretherton-Watt D 171
Brett L 1937
Brewster DH 41, 637
Author index to Volume 85
Key to abbreviations:
(BR) Book reviews; (L) Letters to the editors
2026
British Journal of Cancer (2001) 85(12), 2026-2034
(c) 2001 Cancer Research Campaign
doi: 10.1054/ bjoc.2001.2201, available online at http://www.idealibrary.com on
Bridle H 953
Brienza S 509
Britten R 902
Brizzi MP 1106
Brodin T 129
Brodowicz T 1764
Brooks LA 1551
Brooks SA 1014
Broome JD 930
Brorson SH 1418 (L)
Brosius M 1372
Brown G 453
Brown J 1289
Brown JE 1137
Brown P 1865
Brown RF 1273
Brugnatelli S 141
Brunelli A 1106
Brunner N 85
Brusic V 1738
Bruzzi P 1106
Bryant PE 1157
Bubb VJ 692
Bucana CD 584
Buccoliero AM 333
Buchmann J 791
Buckels J 1865
Budach W 1233
Budinsky A 1764
Budinsky AC 1850
Budman DR 1403
Buhr H-J 1540
Buley I 1014
Bundred NJ 1522
Bunyan DJ 1486
Buring JE 962
Burke D 1640
Burn J 166
Burnett RD 453
Burnstock G 1759
Burrell HC 225
Butler AR 1794
Butow PN 1273
Buzard GS 247
Byrne P 1206
Caceres E 966
Cafferata MA 1452
Calista D 1304
Calistri D 845
Cals L 649
Camlett I 1124
Canamero M 261
Cancer Research
Campaign PK/PD
Technologies Advisory
Committee 1085
Capocaccia R 785
Cappellen D 1515
Carayon P 1771
Carcenac M 1787
Carlsson P 497
Carmichael J 137 (L), 1865
Carnochan P 1640
Carrie C 1646
Carroll-Pankhurst C 1295
Carter N 171
Carton E 1499, 1781
Casadei S 845
Casas A 279, 1794
Caselmann WH 697
Cassidy J 1492
Cassiman JJ 98
Castillo M 966
Catchpoole DR 1564
Cattan N 1412
Certa U 107
Chaggar R 422
Chamberlain M 1175
Chandraratna RAS 453
Chang A 393
Charlton RG 576
Chau I 1258
Chauvin F 816
Chauzy C 1412
Chavaudra N 2017
Chazal M 439
Cheadle JP 1226
Chemotherapy
Standardisation Group of
the United Kingdom
Children's Cancer Study
Group 23
Chen C-J 658
Chen J 1047
Chen Y-L 1185
Chenevix-Trench G 687,
1351
Cheng KK 1667
Cherubini A 333
Chetia CK 661
Cheung N-KV 182
Chevreau C 1467
Chiba T 1006
Chieffi O 333
Chilvers CED 1667
Chio C-C 1185
Cho M 563
Choma D 14
Chopin DK 1515
Chott A 1462
Choudry GA 1137
Chow W-H 1317
Christensen M 568
Christodoulakis M 798
Chughtai S 531
Cimerman N 1193
Cippitelli M 1914
Clark AB 568
Clark TG 944
Clarke PA 692
Clausen OPF 692
Clifford SC 1226
Cocker HA 1746
Colantoni A 1607 (L)
Coleman D 803
Coleman DA 1289
Coleman MG 1486
Collins S 787
Colston KW 171
Comba P 379
Connors T 1421
Connors TA 1070
Conte PF 1452
Cook AM 1624
Cook NR 962, 984
Cook-Mozaffari P 1667
Coppes RP 1055
Coradini D 795
Cornelisse CJ 1347
Corner J 1853
Corrao G 1700
Correale P 1722
Cortesi L 845
Costa J 692
Cotter TG 115
Cottu PH 1240
Coulon V 1467
Courbon F 1467
Cox G 863
Crepin M 917
Crevenna R 1850
Crichton DN 1878
Croke DT 692, 1812
Crolla J 705
Crook T 1551
Cruickshank ME 242
Cruickshank NR 692
Cull A 166, 1842
Cummings M 1351
Cunningham D 692, 1258
Curnow RT 152
Curran S 1492
Curtin JF 115
Curtin NJ 764
Cushing GL Jr 683
Cuvier C 1240
Cvitkovic E 509
Cyjon A 504
Czarnocka B 875
Dabizzi S 333
Daher A 1515
Dai Q 372
Dalgleish AG 473
Dallegri F 463
Daly JS 1499
Dame MK 1600
Dampier K 618
Damstrup L 1211
Danesi R 1425
Daniel F 1865
Danova M 141
Dapino P 463
Darmoul D 772
Daures J-P 14
Davies EJ 1094
Davies MM 1640
Day NE 1667
De Angelis P 692
de Bock GH 1347
de Boer AC 1099
de Bruin R 1206
de Campos JM 204
De Giorgio M 1606 (L)
de Goeij AFPM 692
de Gramont A 509
De Greef C 1387
de Greve J 1
De Greve J 1592
de Jonge MJA 1124
de La Salmoniere P 1883
de Lange Davies C 1968
De Maria N 1607 (L)
de Roquancourt A 1240
de Schipper FA 398
de Vries EGE 1627
de Wasch G 1
de Wit R 1099
de Witte JH 85
de Wolf C 1251
Deakin D 29
DeAngelis P 1900
Dearnaley D 1853
Debled M 1467
Decensi A 1838
Decker HJ 697
Degardin M 649
Del Bufalo D 1914
Del Tacca M 1425
Del Vecchio MT 1722
Delabie J 1900
Delaunay M 1467
Delia D 836
Della Torre G 836
Dellian M 1655
Demicheli R 490
Den Hollander JC 1557
Denic S 1675
Denys H 98
DeRenzo EG 1811 (BR)
Deutsch E 2017
Devaud H 772
Devilee P 1347
Devitt G 1372
Dewar JA 637
Dexter DW 735
Dhom G 972
Di Benedetto M 917
Di Venosa G 279
Diamandis EP 190, 220, 393
Diederichs S 1168
Dienes HP 697
Dillon D 692
Ding L 431
Ding S 1175
Dische S 1113
Dix BR 692
Dogliotti L 1106
Doherty VR 41
Dohlsten M 129
Doki Y 412
Dong C 997
Donghi R 836
Dore J-F 518
Dork T 850
Dorr FA 1753
Dorssers LCJ 538
Dorvillius M 1787
Double JA 1137
Doubrovsky A 157
Douglas F 166
Douglas ML 608
Doyon F 1664
Drabkin HA 64
Dragan C 1546
Drazan KE 584
Drouant GJ 266
Author index 2027
British Journal of Cancer (2001) 85(12), 2026-2034
(c) Cancer Research Campaign 2001
du Vivier A 803
Duarte-Rojo A 1396
Dubost J-J 518
Dubreuil A 439
Ducreux M 509
Duda DG 431
Dumontet C 902
Duncan I 1175
Duncan P 1894
Duncan R 1070
Dunlop MG 1937
Dunn SM 1273
Dunne B 1551
Durand RE 1577
Durham J 453
Durocher F 36
Durrant S 741
Dworkin MJ 1640
Dzik-Jurasz ASK 1624
Earl H 29
East SJ 764
Easton DF 36
Ebihara Y 1706
Eccles DM 1486
Echner H 1527
Edwards DR 55
Edwards J 1894
Edwards JG 863
Edwards S 36
Edwards SM 1383
Eeles R 1383
Eeles RA 36
Eguchi K 9
Ehya H 1952
Eisen T 953
Eisenbrand G 1585
Eissa S 1037
El-Abbadi M 285
El-Badawy SA 1037
Elder JT 1600
Ellis IO 225, 1958
Ellis LM 584
Ellison D 705
Elmquist WF 1987
Elsaleh H 827
Elsby B 1332
ELYPSE Study Group 816
Encio I 1400
Endlicher E 1572
Endoh Y 199
Engels EA 1295, 1298
Engemann H 1801
Engesaeter BO 1968
Enomoto N 228
Enomoto T 247
Epstein AL 463
Eriksson JG 1680
Ernst E 781 (L)
Espenan GD 266
Espie M 1240
Etienne MC 439
European Lung Cancer Working
Party 1444
Eusebi V 422
Evans A 1289, 1551
Evans AJ 225
Evans D 1551
Evans JD 1865
Evans WK 1280
Evoy D 1781
Extra JM 1240
Fabbri A 1535
Fabbro D 2010
Faber C 692
Faithfull S 1853
Falette N 902
Fan Z 303
Fang M 303
Farinati F 1606 (L)
Farnsworth ML 1564
Farrell PJ 1551
Farrugia D 137 (L)
Fava S 141
Fayers P 1478
Fears T 1304
Ferbeye L 46
Ferguson J 139 (L)
Fernandez Garrote L 46
Fiebig H-H 1585
Fiebiger W 1462
Fiehn W 1193
Fieret JH 1359
Figer A 523, 1368
Finkelstein SD 692
Finn Bladder Group 552
Finnegan NM 115
Fiorentini S 1606 (L)
Fioretta G 1251
Fischel JL 439
Flanagan W 1280
Flannigan GM 1137
Flavell KJ 350
Flex D 523, 1368
Florin MC 1444
Florl AR 1887
Flower MA 1640
Focan C 1
Foekens JA 85, 538, 1359
Foley DA 273
FONICAP 1452
Font A 692
Forman D 1322
Formanek M 1462
Forsberg G 129
Forsen T 1680
Forsyth PA 55
Foster TH 727
Foulkes W 1383
Fowler CJ 823
Fox JC 692
Franceschi S 46, 341
Francini G 1722
Francis-Thickpenny KM
687
Franco AV 1564
Francois E 509
Frascona V 2017
Fredericks S 1759
French ME 1504
Fried S 1611
Friedman E 523, 1368
Friery OP 625
Frigerio S 836
Frisch M 1298
Frost T 803
Fruhwald MC 1801
Fryer AA 1504
Fujikawa Y 78
Fujimori T 122, 692
Fujimoto J 313
Fujita M 247
Fujiwara Y 412
Fukuda H 279, 1794
Fulford L 422
Funke PJ 1130
Furuse K 939
Gabra H 944
Galanis E 1432
Galdos O 966
Galisson F 1883
Gallagher R 625
Gallus S 341
Galmarini CM 902
Galteland E 1900
Gamble P 1289
Gamelin E 509
Ganapathi R 747
Gao WM 235
Gao Y-T 372, 1695
Garambois V 1787
Gardaneh M 1564
Garvican L 1289
Gasali PG 1535
Gasco M 1551
Gatta G 785
Gatter KC 261, 881
Gaucherand M 1944
Gaudernack G 692
Gaulier A 1831
Gavish M 1771
Gazdar AF 1510
Gebhardt BM 266
Gedouin D 649
Geisler J 147
Geisler S 74
Geissler F 697
Gelderblom H 1124
Gemmill RM 64
Generali D 1106
Gentet JC 1646
Genus T 531
Georgoulias V 798
Gerald WL 182
German Cooperative Renal
Carcinoma Chemo-
Immuno-therapy Trials
Group 1130
Gescher A 618
Geva R 523, 1368
Ghanem MA 1557
Ghesquiere H 816
Giaccone G 1456
Gianni S 1606 (L)
Giardina G 141
Giaretti W 692
Giatromanolaki A 881
Gibbs A 787
Gil M 1400
Gil-Diez-de-Medina S 1515
Gilbertson R 705
Gilmore AP 1522
Giner V 1444
Gioanni J 1412
Giordano M 141
Giorgi G 1722
Giovannucci E 346
Giuliano AE 1340
Given-Wilson R 171
Glaesener R 692
Glanzmann C 2010
Glaser B 36
Glaussel F 1787
Gleig A 1157
Glennie MJ 1619
Glimelius B 1265
Glover C 1640
Gnanapragasam VJ 1928
Gnant M 1850
Gneiting T 1655
Goedert JJ 337, 1295, 1298
Goemann C 546
Goetz AE 1655
Goh H-S 692
Goh L-b 1175
Gohring B 924
Going JJ 1894
Goldbohm RA 977
Goldgar D 1383
Goldstein AM 527
Gong Y 1706
Gonzalez-Gomez P 204
Goodwin LO 930
Gordon T 493, 831
Gore ME 953
Gough AC 1486
Gould A 1842
Gould S 831
Graham-Brown R 803
Gramatzki M 1130
Grasso D 141
Gratio V 772
Grazia Daidone M 795
Greaves MJ 293
Grebenchtchikov N 85
Greenberg ER 683
Greif F 504
Griebel J 1655
Grieu F 827
Griffin M 1781
Grigor KM 1894
Grimm J 791
Gritzapis AD 1527
Groft LL 55
Gronchi A 1535
Groves FD 337
Gualde N 1467
Gubala E 875
Guenther U 1540
Guerrero I 966
Guerrieri-Gonzaga A 1838
Guerts-Moespot A 85
2028 Author index
British Journal of Cancer (2001) 85(12), 2026-2034 (c) Cancer Research Campaign 2001
Guevara P 1396
Gustafsson B 1871
Gusterson B 1551
Haber M 1564
Haboubi NY 787
Haddad G 1219
Hadjiloucas I 1522
Hainaut P 721
Haites NE 242, 1492
Hajian-Tilaki KO 1671
Hakansson A 1871
Hakansson L 1871
Hakulinen T 357, 367
Halgunset J 1968
Hall CN 692
Hall DMS 1014
Halter SA 692
Hamilton SR 1037
Hamilton Stewart PA 1137
Hammond LA 453
Hamoudi RA 1383
Hansen LRT 568
Hansson E 552
Hara N 557, 1119
Harada M 9
Hardoff R 504
Harita S 9
Harland SJ 823
Harmey JH 273
Harris A 78
Harris AL 881
Hasegawa J 1510
Hasegawa K 939
Hasegawa Y 102, 1634
Hashida H 1922
Hashimoto I 93
Haskill J 668
Hassan M 2004
Haug I 1968
Hausmann F 1572
Hawkins N 692
Hawkins R 140 (L)
Hedelin G 808
Heemann U 1047
Hegyi I 2010
Heinzel S 1527
Hejna M 1764
Heldin P 600
Heliovaara M 357
Helle SI 74, 147
Hemmi H 1064
Hemminki K 997
Hennis B 1124
Henzen-Logmans SC 1359
Herbst H 1540
Hernan R 705
Herrero R 46
Hess C 2010
Heuvel JJTM 85
Heuze A 764
Hicklin DJ 584
Hickson ID 261
Hida Y 1922
Higgins C 608
Hilakivi-Clarke L 1680
Hill C 1664
Hill L 493
Hill M 1258
Hino S 122
Hirakawa K 612
Hiraki S 9
Hirano T 1706
Hirao Y 563
Hiraoka K 1922
Hirata K 752
Hirst DG 625
Hjelmqvist B 1871
Ho C-K 658
Ho JWC 692
Hobbs SM 1746
Hocker JE 1564
Hoffman K 1265
Hoffmann I 692
Hofstra LS 1627
Hogg D 527
Hollis R 23
Holly JMP 74, 147
Holm L-E 362
Holmberg E 362
Holmberg L 346, 869
Holte H 1900
Hongo A 93
Hoon DSB 1340
Hopfner M 1771
Hopwood P 137 (L), 166
Hoque MO 122
Horton C 325
Hoshai H 1064
Houlihan PS 1037
House A 1842
Howe K 1824
Howells LM 618
Howie AJ 152
Hsieh C-C 984
Hsu H-K 658
Hsu T 1219
Hubert A 36
Huddart R 213, 1853
Huddart RA 1624
Hudes GR 735
Hudson EA 618
Hughes CM 625
Huisman C 1456
Hunt D 705
Hunt NCA 78
Hunter JAA 803
Huot TJG 836
Hupp TR 1102, 1878
Huusko P 209
Huynh KT 1340
Hynes NE 1787
Iacopetta B 827
Iacopetta BJ 692
Ikeda N 1706
Illiger HJ 1130
Imamura M 1006
Imrie CW 1865
Inai M 1006
Ingvar C 674
Inoue H 713
Inoue S 1731
Irmin L 523, 1368
Isbert C 1540
Ishihara S 741
Ishikawa T 612
Ishikawa Y 741
Isla A 204
Islam-Ali B 758
Isola JJ 1201
Issa J-P 930
Italian Lung Cancer Task
Force (FONICAP) 1452
Itoh T 1922
Itonaga I 78
Izumi N 228
Jabbour HN 1023
Jackman AL 446
Jager U 1462
Jaiswal AS 898
James ND 152
Jan P 1664
Jandik P 692
Janin A 1883
Janota-Bzowski M 875
Jansen A 1771
Jansen SE 1472
Jarvinen R 357
Jarzab B 875
Jaunin P 1713
Jayson GC 1094
Jefferies S 1383
Jin F 372
Jin Z 69
Jodrell DI 1937
Johannsson O 1201
Johansson H 1838
Johns LE 1289
Johnson CD 1865
Johnson PWM 1619
Johnson R 649
Johnsson A 1206
Johnston PG 1937
Johnston RN 55
Jones C 422
Jones EL 453
Jones J 152
Jones JL 863
Jones PW 1504
Jones-Wallace D 705
Jordhoy MS 1478
Joseph D 827
Jozefonvicz J 917
Juji T 741
Jullian E 692
Jung K 220, 393
Jung YD 584
Kaasa S 1478
Kaasinen E 552
Kabanov AV 1987
Kadouri L 36
Kajita T 255
Kakolyris S 798
Kalbakis K 798
Kamei H 9
Kameshima H 752
Kampinga HH 1055
Kandioler D 1764
Kang H 1147
Kano M 1006
Kao E-L 658
Karagas MR 683
Karjalainen-Lindsberg M-L 383
Karlsson P 362
Karstens JH 850
Kasahara T 557, 1119
Kasamon YL 1738
Kashiwaba M 69
Kato H 1706
Kato Y 1706
Katoh H 1922
Katoh K 1922
Katsaros D 190
Kawabata W 1731
Kawada Y 563
Kawahara M 939
Kawamata H 122
Kawano K 247
Kawasaki H 1510
Kawasaki T 557
Kayhty H 337
Kayitalire L 649
Kayser K 1193
Keeling PWN 1499
Kelland LR 1746
Kelleher D 1499
Kelsey A 831
Kemp EH 875
Kempski H 493
Keohavong P 235
Kernohan N 1878
Kernohan NM 1102
Khan S 758
Khatun S 313
Kherfellah D 1077
Kierstead LS 1738
Kikuchi A 741
Kim H 1147
Kim S-C 563
Kim SE 1147
Kimura F 1887
Kimura K 255
Kimura W 199
Kirchner H 1130
Kirkwood JM 1738
Kirsten ras In-Colorectal-Cancer
Collaborative Group 692
Kishi K 412
Kishimoto Y 1510
Kiura K 9
Klaassen I 630
Klastersky J 1444
Klein G 1952
Klein Kranenbarg E 780 (L)
Kleinerman ES 747
Klevenvall L 2004
Klijn JGM 85, 538, 1359
Klinzig F 808
Klug S 966
Klumpen S 1168
Knauper V 55
Author index 2029
British Journal of Cancer (2001) 85(12), 2026-2034
(c) Cancer Research Campaign 2001
Knekt P 357
Knowles MA 1887
Knuchel R 1572
Knuechel R 727
Kobayashi D 752
Kodama J 93
Kodama T 431
Koduru P 930
Koelbl H 791
Koestler W 1764
Koezuka Y 741
Kogel D 1801
Kogner P 2004
Koi M 1064
Konaka C 1706
Kondo S 1922
Konings AWT 1055
Konishi N 563
Koopman FJ 1472
Kopchick JJ 428
Kopitar G 1193
Kopp-Schneider A 1372
Koren G 1611
Kortmann RD 1233
Kos J 1193
Kote-Jarai Z 36
Koukourakis MI 881
Koumakis G 1444
Kourbali Y 917
Kouroussis Ch 798
Kozikowski AP 1771
Kraemer M 917
Krainer M 1764
Kreis W 1403
Kricker A 668
Krieg R 1572
Krtolica K 692
Krul MRL 1137
Krysander L 1871
Kudo T 93
Kudoh S 939
Kugler K 1168
Kulkarni S 325
Kuma K 102
Kunkel TA 568
Kunstfeld R 1764
Kurita Y 1326
Kurtaran A 1462
Kusak ME 204
Kushi LH 372
Kusterer K 1546
Kyriakopoulou L 190
La Vecchia C 341, 1700
Laburthe M 772
Lachlan G 1322
Lafitte JJ 1444
Lafleur MA 55
Lagergren J 1317
Lagrange J-L 1412
Laguerre B 649
Lakhani SR 422
Landi G 1304
Landi MT 1304
Lando PA 129
Lang SH 590
Langdon SP 1753
Lange D 875
Langlois R 1787
Lantieri F 836
Lantzsch T 791
Larroque Ch 1787
Laslo D 1611
Latz J 649
Laube F 924
Launonen V 209
Laurence V 1240
Laurent-Puig P 692
Le Deley MC 1646
Le Frere-Belda MA 1515
Le Pelletier F 1883
Leach MO 1085
Lebitasy MP 808
LeCesne A 816
Lecomte J 1444
Lee C-J 658
Lee I-M 962
Lee J-M 658
Lee JQ 692
Lee JS 1147
Lee L 1289
Lee MC 285
Lee Y-C 658
Lees N 692
Lefebvre JL 649
Leirvaag B 147
Leitch A 1937
Lence Anta J 46
Lerebours F 1240
Lessells AM 1937
Leung HY 576, 1928
Levi F 341
Levi F 509
Levin B 1037
Levine PH 337
Levrat C 902
Lewis R 137 (L)
Leydon G 641
Leyvraz S 1713
Li C 98
Li S 1987
Li Y 600
Liestol K 1418 (L)
Lin S-J 1185
Lin VC-L 1978
Lindblad P 984
Lishner M 1611
Lister TA 29
Liston J 1289
Liu B 303
Liu L 527
Liu S 1335, 1504
Liu W 584
Liu XM 1403
Lo Vullo S 1535
Lobelle JP 1
Lock RB 1564
Locker GJ 1850
Logan DM 1280
Logan RFA 1667
Loge JH 1478
Loizidou M 1759
Lomas J 204
Lombard-Bohas C 721
Longley DB 1937
Loni L 1425
Loning T 546
Lonning PE 74, 147
Look MP 85, 1359
Loos WJ 1124
Lorite MJ 297
Los G 1206
Losi L 692
Louhelainen J 1887
Love DR 687
Love SB 29
Lovig T 692
Lozzi L 1722
Lu C-M 984
Lu Y 303 (two)
Lu Y-J 213
Lubin R 1883
Ludwig C 1764
Luketich JD 235
Lukish JR 1037
Lund Nilsen TI 959
Lund P 697
Lundell M 362
Lunec J 705
Lung Cancer Working Group 939
Luo L-Y 220
Luoto R 1680
Luscombe CJ 1504
Maas S 1887
Maaser K 1771
McCauley RA 735
McConville CM 531
McCready VR 1640
McDowell H 831
McDowell HE 1878
McFadyen MCE 242
McFarlane G 1322
McIntosh RS 875
McKay JA 1492
McKeage MJ 1219
McKeown SR 625
McKinney PA 1667
McLaren A 1175
McLeod HL 242, 1492
McManus A 831
McManus R 1499
McPherson K 166, 641
McPhillips F 1753
Mady HH 235
Maehlen J 1418 (L)
Maggiorella L 2017
Magois C 1831
Mahanta J 661
Maher ER 1226
Maitland NJ 590
Majois F 1
Mak I 953
Makke J 1240
Malik S 692
Malingre MM 1472
Mamalaki A 1527
Mammen A 1952
Mancini M 463
Mann JR 350, 531
Manno M 1607 (L)
Mansi JL 171
Manson MM 618
Mantel MA 1124
Mantyniemi S 209
Mao Y 1335
Mapp T 831
Marangoni E 2017
Marchetti S 439
Mari B 1412
Mariani L 1535
Marie J-C 772
Marko D 1585
Marolleau JP 1240
Marsden RA 803
Marsh S 1492
Marshall GM 1564
Marsili S 1722
Martel-Planche G 721
Martinez-Merino V 1400
Marty M 509, 1240
Marumo F 228
Mashino K 713
Massey A 1258
Matsui K 939
Matsuno S 431
Mattsson A 362
Maughan TS 1422
Mavroudis D 798
Mayeur D 1240
Maynard JH 1226
Mazeau C 1412
Mech P 1801
Mechler C 1831
Mechniaud F 1646
Mehta J 325
Meijer C 966
Meijer CJLM 398
Meijer-van Gelder M 538
Meijers-Heijboer H 538
Melhem MF 235
Melia J 641, 656, 803
Meling GI 692
Melvin WT 242
Menard S 1964
Mende T 791
Merrett S 422
Merry CLR 1094
Messmann H 1572
Methner C 546
Metzger R 1168
Metzner B 1130
Meyer L 1853
Mezzelani A 405, 1535
Michalski A 493
Michel J 1444
Micheli L 1722
Michon J 1646
Miclea JM 1240
Migliaccio M 1400
Milano G 439
Milde-Langosch K 546
Miller DW 1987
Miller ID 242
2030 Author index
British Journal of Cancer (2001) 85(12), 2026-2034 (c) Cancer Research Campaign 2001
Miller K 1759
Milliken D 891
Mills GB 303
Milne SA 1023
Minami H 1634
Miodini P 795
Misawa H 1326
Mitra S 727
Miura N 1510
Miyamoto M 1922
Miyasaka Y 228
Miyata H 412
Miyauchi A 102
Moch C 1883
Mocroft A 1824
Mokhtar N 1037
Moller M 1168
Moller TR 674
Mommen P 1444
Monden M 412
Monia BP 1753
Monks NR 764
Montanari G 141
Montaudon D 1077
Moon SE 1600
Mora J 182
Moreau L 808
Morelli D 1964
Morgan B 115
Mori M 713
Moriya T 1731
Morris A 297
Morris MB 1998
Morrissey C 1226
Mortimer EA Jr 1295
Morton D 692
Moss S 656, 803
Moss SM 1289
Mossman J 641
Motoyama T 69, 199
Mott LA 683
Moynihan C 641
Moysan A 1883
MPT Collaborators 1383
Mucci LA 678
Muehlbacher F 1850
Mulder NH 1627
Mullen P 1753
Muller O 692
Muller-Tidow C 1168
Mulligan E 1781
Multani AS 898
Munoz N 46, 966
Muramatsu M 1731
Murata Y 247
Murawaki Y 1510
Murdoch J 1824
Murphy KM 1564
Murray GI 242, 1492
Murray MM 625
Murray PG 350
Muzik H 55
Mycroft J 23
NACCP Group 1437
Nagashima H 713
Nagayama K 228
Nakagawa H 412
Nakakubo Y 1922
Nakamoto S 1510
Nakashiro K 122
Nakayama T 1326
Narayan S 898
Nardini E 1964
Naylor B 1137
Neal DE 576, 1928
Negri E 341
Nelson P 350
Neoptolemos JP 1865
Neri P 1722
Neumaier C 1452
Neuman-Levin M 504
Newell DR 764
Neyns B 1592
Neyroud I 1251
Ng EH 1978
Ng EH-L 1978
Nguyen Bui B 1467
N'Guyen Bui B 1646
Nicholson RI 1958
Nicol A 741
Nicol DL 608
Nicoll G 1878
Nicolson M 137 (L)
Nieda M 741
Niederberger E 1585
Nieminen P 552
Nierenberg DW 683
Niitani H 939
Nijman RJM 1557
Niketeghad F 697
Ninane V 1444
Nishihara T 612
Nishino H 612
Nishioka K 412
Niyikiza C 649
Nobrega KM 1280
Noci I 333
Noda K 1064
Nonni AV 422
Noordzij MA 1557
Nordgren H 869
Nordin K 1265
Norman A 1258, 1624
Norman AR 692
Norris MD 1564
Nulman I 1611
Nyren O 678, 1317
Oates J 692
Obara K 557, 1119
Oberlin O 1646
O'Byrne KJ 473, 863
Oda M 255
O'Donnell M 576
O'Donoghue DP 692
O'Dorisio MS 266
O'Dorisio TM 266
Ogawa S 1731
Ohira M 612
Ohira T 1706
Ohkusa T 692
Ohlsson L 129
Ohno Y 1634
Ohta Y 255
Okimoto N 9
Okuda H 93
Olaitan A 1824
Olschwang S 692
Olson W 1738
Olsson H 674
Omotehara F 122
Omura K 692
On On Chan A 1037
O'Nions J 1551
Ono Y 692
Onuki N 1510
Ormberg IW 1968
Orntoft TF 568, 1211
Orosz C 285
Ortiz Reyes RM 46
Osakabe M 69
Oshikiri T 1922
Oshimura M 1510
Osin P 213
Osmond C 1680
Osterreicher C 1462
Oswald J 1289
Ott RJ 1640
Ottonello L 463
Oude Luttikhuis MEM 531
Ouwehand M 1472
Ouyang N 1047
Ozaki K 247
Padhani AR 1624
Padovan E 107
Paesmans M 1444
Page A 1289
Pageaux J-F 1944
Paglierani M 333
Pahernik S 1655
Paik Y-K 1147
Papamichail M 1527
Papelard H 1347
Papini D 405
Park JH 1147
Parker C 1958
Parkes SE 350
Parkin D 242
Parmiani G 836
Parry L 1226
Pasini B 836
Pastuszko D 875
Patel A 1226
Pathak S 898
Patnick J 1289
Patterson LH 625
Patzelt T 1130
Paulsen T 74
Pauly M 692
Pavillard V 1077
Pawelec G 1527
Pearson A 705
Peaston AE 1564
Pedersen MW 1211
Pedrotti C 141
Peguet-Navarro J 1944
Pelegrin A 1787
Pellizzaro C 795
Peng H 930
Pensotti V 845
Penz M 1462
Pera MF 608
Peretz T 36
Perey L 1713
Perez SA 1527
Perng R-P 1247
Perotti C 1794
Perret G 917
Perry R 705
Pesatori A 1304
Peters G 836
Peters R 1713
Petersen S 1372
Petrioli R 1722
Pezzella F 881
Philip T 1646
Phillips RM 1137
Phukan RK 661
Picton S 23
Pidgeon GP 273
Pierotti M 845
Pierotti MA 405, 836, 1535
Pilotti S 405, 1535
Pinder SE 225, 1958
Pinkerton CR 1746
Pinkerton R 831
Piris J 692
Pivot X 649
Plouet J 1944
Poli MA 141
Pollak M 428
Pont J 1462
Ponthan F 2004
Poon R 98
Poppe M 1801
Porrata LF 484
Portengen H 538
Postmus PE 1456
Potter JD 372
Poulsen HS 1211
Powell JE 350, 531
Powles R 325
Pozzessere D 1722
Prang J 1372
Preece PE 1157
Prehn JHM 1801
Prevost G 115
Price O 29
Price P 1085
Price SA 758
Pricolo VE 692
Primrose JN 1486
Pritchard-Jones K 831,
1746
Probst-Hensch NM 1695
Prochilo T 1452
Pruschy M 2010
Pugliese P 141
Puisieux A 902
Pujol JL 14
Pulimood AS 1928
Puolakkainen P 383
Author index 2031
British Journal of Cancer (2001) 85(12), 2026-2034
(c) Cancer Research Campaign 2001
Purdie CA 1102
Purdie D 1351
Purohit A 808
Pyle L 953
Qi K 1340
Qian L 930
Qiao Z 1332
Qiu WM 176
Quantin X 14
Queneau P-E 1251
Quirke P 692
Quoix E 808
Rabes HM 692
Raderer M 1462
Rades D 850
Radford J 29
Radice P 845
Radvanyi F 1515
Raitanen M-P 552
Rajpert-De Meyts E 220
Raju S 891
Rakowsky E 504
Ramani P 531
Rampaul RS 1958
Rampling D 493
Rampling R 1810 (BR)
Ranganathan S 735
Ranieri E 1738
Ranson M 909
Raouf A 1781
Rapallo A 692
Rath HC 1572
Rathi A 1510
Ravaud A 1467
Ray-Coquard I 816
Raymond E 649
Raymond L 1251
Reddy A 1551
Redfern D 350
Rees SA 531
Reimertz C 1801
Reinmuth N 584
Reitz M 1130
Remick DR 1600
Rettrup B 1871
Rewcastle NB 55
Rexrode KM 962
Rey JA 204
Reynolds JV 1499, 1781
Ricca A 1914
Riccardi A 141
Ricci P 379
Richard S 1077
Richardson DM 687
Riches AC 1157
Rieck G 546
Riecken E-O 1540, 1771
Riesterer O 2010
Rigg A 325
Rinaldi E 141
Rinnab L 1372
Rintala E 552
Ripoche V 649
Rissanen H 357
Ritchie AA 1753
Riva C 405, 1535
Rivera E 1396
Robert J 1077
Roberts MS 157
Robertson AJ 1157
Robertson JFR 1958
Robson CN 1928
Rochet N 1412
Rodolfo M 836
Roesch-Gateau O 902
Rogers L 1794
Rognum TO 692
Rohatiner A 29
Rolhion C 518
Rooney PH 1492
Rosell R 692
Rosenberg PS 1298
Rosing H 1472, 1627
Ross RK 1695
Rossi B 1412
Rosso R 1452
Roth T 1585
Roufogalis BD 1998
Rovini D 836
Rozendaal L 398
Rozycka M 1551
Rubin G 1289
Rudas M 1764
Rudin C 325
Rumio C 1964
Rummele P 1572
Runsink AP 398
Russell S 1432
Russo A 692
Ryan BM 1499
Rylander E 1153
S-1 Cooperative Study
Group 939
Saarialho-Kere U 383
Sabatino M 1722
Sabokbar A 78
Sadanaga N 713
Sadetzki S 1368
Saga S 563
Sagawa M 1326
Saint Frison M 1831
Saito K 1119
Saito Y 1032
Sakaguchi H 313
Sakai S 1634
Sakata K 69
Sakurai T 1006
Salamon E 1
Sallstrom J 1153
Salmela MT 383
Saltnes T 1478
Sampson JR 1226
Sandblom G 497
Sann H 924
Santos C 966
Sappino AP 1251
Sapunar F 953
Sarasa JL 204
Sasaki T 255
Sasano H 1731
Saso R 325
Satchi R 1070
Sato C 228
Sato K 199
Sato M 122
Sato S 1032
Saunders MI 1113
Saurin JC 721
Sauter ER 1952
Sawada T 612
Saxby MF 1504
Scheidtmann KH 1801
Schellens JHM 1472
Scherubl H 1771
Schirmacher P 697
Schmidinger M 1850
Schmidt C 741
Schmitt C 1646
Schmitt D 1944
Schneider M 649
Schneider PM 1168
Scholmerich J 1572
Schot ME 1472
Schott H 1130
Schradin T 1372
Schramel FMNH 1456
Schulte K 1585
Schulz WA 1887
Schumm-Draeger PM 1546
Schuppan D 1540
Schuyer M 1359
Sciot R 98
Scoazec JY 721
Scorilas A 190
Scott D 293
Scott N 787
Scottish Cancer Therapy
Network 637
Scotton C 891
Screnci D 1219
Sculier JP 1444
Sebban C 816
Segawa Y 9, 939
Seifeldin IA 1037
Seifert H-H 1887
Seiler M 107
Seki N 93
Sekirnik A 1193
Selby P 1842
Semenciw R 1335
Senagore AJ 692
Serve H 1168
Sette A 1738
Seyfried TN 285
Sfondrini L 1964
Shah TK 1137
Shaheen RM 584
Shamma A 412
Shankar A 1759
Shaper AG 1311
Sharkey I 23
Sharp L 1667
Sharrard RM 590
Shaw D 129
Shelling AN 687
Shen N 930
Shennan MG 527
Shi R 991
Shibayama T 9
Shibuya K 431
Shimada Y 1006
Shimamura H 431
Shimokata K 1634
Shinohara T 1922
Shiozaki H 412
Shipley J 213, 831
Shou Y 1706
Shtoyerman-Chen R 1368
Shu X-O 372
Sidiropoulos M 393
Sidney J 1738
Siebert GA 157
Siebrands C 850
Siegfried JM 235
Sigsjo P 497
Simon J 1372
Simonato L 341
Singhal S 325
Sinha D 337
Sirohi B 325
Sitas F 1322
Sivridis E 881
Skawran B 850
Slingluff CL Jr 1738
Sliutz G 791
Smalley K 953
Smedshammer L 1900
Smeets SJ 630
Smeland EB 1900
Smit EF 1456
Smith A 1842
Smith DR 692, 1812
Smith HJ 297
Smith J 1824
Smithers BM 157
Smyth JF 944, 1753
Snary D 692
Snijders PJF 398
Snow GB 630
Sobin LH 780 (L)
Soliman AS 1037
Soliman EH 1557
Song E 1047
Sotelo J 1396
Sotiriadou R 1527
Sotiropoulou PA 1527
Soufir N 1883
Souglakos J 798
Sozzi G 405, 1535
Spagnoli GC 107
Sparreboom A 1099, 1124
Spencer SK 683
Spiro SG 1609 (BR)
Spreadborough A 293
Staff S 1201
Stanczyk FZ 1695
Staquet M-J 1944
Stark A 1865
Stark M 590
Starzec A 917
Stassar MJJG 1372
2032 Author index
British Journal of Cancer (2001) 85(12), 2026-2034 (c) Cancer Research Campaign 2001
Stebbing J 953
Steel CM 1157
Steel J 1265
Steen HB 1900
Steger GG 1850
Stegmaier C 972
Steininger R 1850
Steinmann D 850
Stern PL 129
Stevenson DAJ 1492
Stewart F 937
Stewart M 1937
Stewart ME 944
Stockton D 41
Stokke T 1900
Stora de Novion H 1412
Storkus WJ 1738
Stoter G 1099, 1359
Straetmans N 1387
Strand A 1153
Strange RC 1504
Stremmer A 1193
Streuli CH 1522
Strickler HD 1295
Strong V 1842
Struewing JP 527
Stumbo M 1535
Sturdy L 1289
Sturmer T 972
Stuttgen MA 608
Stylianou K 798
Su F 1047
Suarez H 1831
Sudaryo MK 1557
Sugimachi K 713
Sugita M 64
Sulkes A 504, 523, 1368
Sullivan A 1551
Summersgill B 213
Sun X-L 930
Sun XF 692
Sunamura M 431
Sutton G 1289
Suzuki T 1731
Suzuoki M 1922
Sweep CGJ 85
Sweneey E 1781
Swerdlow AJ 1332
Taal BG 1437
Tabata M 9
Tabone E 902
Taddei GL 333
Tadokoro K 741
Tagnon A 1
Takahashi K 557, 1119
Takano T 102
Takata I 9
Takeda K 431
Takigawa N 9
Talks K 881
Tamaya T 313
Tamborini E 405, 1535
Tamir A 1368
Tammela TLJ 552
Tamura G 69, 199
Tamura M 255
Tamura S 412
Tan MG-K 1978
Tanaka F 713
Tanaka M 692
Tanaka N 64
Tanaka Y 255
Tang WC 1585
Taniere P 721
Tanimoto M 9
Tanner MM 1201
Tattersall MHN 1273
Taylor I 1759
Tchekmedyian S 152
Tchirkov A 518
Teas J 372
Tejpar S 98
Ten Bokkel Huinink WW 1472
Teraoka H 612
Terrier P 1646
Terry P 346
Teugels E 1592
Thatcher N 137 (L)
Thebo JS 692
Theodor L 1368
Thiery JP 1515
Thiriaux J 1444
Thomas V 171
Thompson AM 1102, 1157, 1878
Thompson JF 157
Thompson MG 297
Thomson CS 637
Thomson JZ 225
Thykjaer T 568
Thykjaer T 1211
Tibaldi C 1452
Tidy JA 1551
Tiffin N 1746
Tinelli C 141
Tisdale MJ 297, 758
Tollenaar RAEM 1347
Tomasic G 795
Tomek S 1764
Tomita Y 557, 1119
Tomkins SE 453
Torrisi R 1838
Tortolina G 463
Traub T 1462
Travis WD 1510
Treleaven J 325
Trinca S 379
Trisciuoglio D 1914
Trotti G 141
Troungos C 692
Tseng WW 584
Tsuchida S 1032
Tsuchiya F 1731
Tsuchiya T 69, 199
Tsukada H 1326
Tsunezuka Y 255
Tucker MA 527, 1304
Tuomilehto J 1680
Turley H 261, 881
Turner R 1340
Twelves CJ 637
Tytgat J 1
Uchida D 122
Ueda M 64
Ueki M 64
Uemura H 563
Ueoka H 9
Ugnat A-M 1335
UK Cancer Cytogenetics
Group 831
UK Childhood Cance
Study Investigators 1685
UK Children's Cancer
Study Group 831
Ukena D 1130
Uleryk E 1611
Umemoto M 1032
Urosevic N 692
Usadel KH 1546
Usel M 1251
Usuba O 199
Vaccarella S 46
Valagussa P 490
Valavanis C 692
Vamvakas L 798
van Belle S 1
Van Camp B 1387
Van Cutsem E 98
Van Cutsem O 1444
van de Ouweland AMW 538
van de Vijver MJ 1347
van den Brandt PA 977
Van den Heuvel MM 1557
van der Burg MEL 1124, 1359
Van der Kwast TH 1557
van der Linden HC 398
van der Zee AGJ 1627
van Ee CC 687
Van Hul W 176
Van Krinks CH 453
van Lier JE 1787
Van Riet I 1387
van Staveren IL 538
van Steenbrugge GJ 1557
Van Tellingen O 1472
van Tienoven ThH 85
Van Tinteren H 1437
Vande Broek I 1387
Vanderkerken K 1387
Vandervellen R 1
Vanier-Viornery A 902
Varani J 1600
Varenhorst E 497
Varley JM 293
Vassy R 917
Vatten LJ 959
Velarde C 966
Velikova G 1842
Venutolo E 1883
Verhoog L 538
Vermeij J 1592
Veronesi G 1838
Verrelle P 518
Verweij J 1099, 1124
Villa E 1607 (L)
Villette JM 590
Vilmer C 1883
Vindevoghel A 1
Virgolini I 1462
Virmani A 1510
Voelter W 1527
Vogt U 1168
von der Linden GHM 1099
von Jurgenson S 1130
von Schlippe M 823
Voorhorst FJ 398
Voorzanger-Rousselot N 902
Vousden KH 1813
Vuong V 2010
Wadler S 692
Wagner J 1295
Wakai K 1634
Wakai S 1326
Walboomers J 966
Walker M 1311
Walker RA 618, 863
Wallace H 23
Waller DA 863
Wallgren A 362
Walsh T 692
Wandert T 1130
Wang G-S 1247
Wang H-Y 1162
Wang J 176
Wang LG 1403
Wang M 1047
Wang ST 692
Wang Y-S 1185
Wang Z-X 1162
Wannamethee SG 1311
Ward R 692
Wardman G 1289
Warin AP 803
Warnberg F 869
Wascher RA 1340
Watanabe G 255
Watanabe M 228
Watanabe N 752
Watanabe T 1037
Watanabe Y 9, 1064
Waters JS 1258
Watson JC 266
Watson M 166
Watson PF 875
Watters AD 1894
Webb A 1258
Webley SD 446
Weeks J 1824
Weetman AP 875
Weiner D 1206
Weinreb M 350
Weisinger G 1771
Weizman A 1771
Welsh SJ 446
Wen B 2017
Wencyk PM 1958
Wenzel C 1850
Werle B 1193
Wessel N 1418 (L)
West AF 576
West J 64
White J 803
Author index 2033
British Journal of Cancer (2001) 85(12), 2026-2034
(c) Cancer Research Campaign 2001
Whitehead SM 803
Wickramasinghe C 705
Wilander E 1153
Wilkinson K 692
Will BP 1280
Willemse PHB 1627
Willhardt I 924
Williams ARW 1023
Wilms E 1124
Wilson A 29
Wilson ARM 225
Wilson J 891
Wilson R 1289
Wiltschke C 1764
Winder R 1289
Wingren S 859
Winn R 64
Winqvist R 209
Winship IM 687
Wloch J 875
Wolf CR 1175
Wolfson MC 1280
Wolk A 346, 984
Woltering EA 266
Wolters M 791
Wonderling D 166
Wong J 1206
Wong NACS 1937
Wood J 2010
Woodman CBJ 787
Workman P 1085
Wright P 1842
Wroughton MA 803
Wu D-C 658
Wu H 176
Wu L 261
Wu M-C 1162
Wu M-T 658
Wuyts W 176
Xiao CY 176
Yagihashi A 752
Yajima T 752
Yamaguchi H 713
Yamamoto H 412
Yamasaki M 247
Yamauchi J 431
Yang B-C 1185
Yang K-Y 1247
Yang P-W 658
Yano M 412
Yashiro M 612
Yasuda T 412
Yates AJ 285
Ye W 678, 1317
Yokoyama A 1326
Yokoyama Y 1032
Yoshida H 102, 122
Yoshida K 563, 1706
Yoshikawa K 563
Yoshimori K 939
Yoshino K 247
Yoshinouchi M 93
Young J 692
Young LS 350
Yu C 98
Yu H 991
Yu MC 1695
Yuan J-M 1695
Yuen ST 692
Zafrani ES 1515
Zanghellini E 1412
Zeegers MPA 977
Zeilstra LJW 1055
Zeinoun Z 1592
Zeitz M 1771
Zellweger M 1251
Zhang G 176
Zhang H 692
Zhang J-C 428
Zhang SZ 176
Zhao F 1706
Zheng W 372
Zhou Z 747
Zhu D 1037
Ziegler H 972
Zielinski CC 1764
Zielinski GD 398
Zietz C 692
Ziyaie D 1102
Zoetmulder FAN 1437
Zoller M 1372
Zupi G 1914
Zweit J 1640
Zwelling LA 747
2034 Author index
British Journal of Cancer (2001) 85(12), 2026-2034 (c) Cancer Research Campaign 2001

				

